# Pantothenate kinase-associated neurodegeneration is not a synucleinopathy

**DOI:** 10.1111/j.1365-2990.2012.01269.x

**Published:** 2012-03-14

**Authors:** A Li, R Paudel, R Johnson, R Courtney, A J Lees, J L Holton, J Hardy, T Revesz, H Houlden

**Affiliations:** * Department of Molecular NeuroscienceLondon, UK; † Queen Square Brain Bank, UCL Institute of NeurologyLondon, UK; ‡ Rita Lila Weston Institute of Neurological StudiesLondon, UK; § Department of Pediatrics, University of MarylandBaltimore, MD, USA

**Keywords:** α-synuclein, Lewy body, neurodegeneration with brain iron accumulation, neurofibrillary tangles, pantothenate kinase 2, tau

## Abstract

**Aims:** Mutations in the pantothenate kinase 2 gene (*PANK2*) are responsible for the most common type of neurodegeneration with brain iron accumulation (NBIA), known as pantothenate kinase-associated neurodegeneration (PKAN). Historically, NBIA is considered a synucleinopathy with numerous reports of NBIA cases with Lewy bodies and Lewy neurites and some cases reporting additional abnormal tau accumulation. However, clinicopathological correlations in genetically proven PKAN cases are rare. We describe the clinical, genetic and neuropathological features of three unrelated PKAN cases. **Methods:** All three cases were genetically screened for the *PANK2* gene mutations using standard Sanger polymerase chain reaction sequencing. A detailed neuropathological assessment of the three cases was performed using histochemical and immunohistochemical preparations. **Results:** All cases had classical axonal swellings and Perls' positive iron deposition in the basal ganglia. In contrast to neuroaxonal dystrophies due to mutation of the phospholipase A2, group VI (*PLA2G6*) gene, in which Lewy body pathology is widespread, no α-synuclein accumulation was detected in any of our PKAN cases. In one case (20-year-old male) there was significant tau pathology comprising neurofibrillary tangles and neuropil threads, with very subtle tau pathology in another case. **Conclusions:** These findings indicate that PKAN is not a synucleinopathy and, hence the cellular pathways implicated in this disease are unlikely to be relevant for the pathomechanism of Lewy body disorders.

## Introduction

Neurodegeneration with brain iron accumulation (NBIA) encompasses several progressive movement disorders with excessive iron accumulation in the brain parenchyma, especially in the basal ganglia. Patients with NBIA can present with symptoms and signs such as ataxia, dystonia, muscle rigidity, dysarthria, retinal or optic degeneration and dementia [1]. Classification of NBIA patients has gradually evolved due to advances in clinical, pathological and most importantly the genetic understanding of these disorders. Earlier literature described several common groups, including the infantile or classical neuroaxonal dystrophy (iNAD), also known as Seitelberger disease, the Hallervorden–Spatz syndrome (HSS) [2],[3] and also late infantile or juvenile forms [4]. iNAD has an onset before the age of 6 years and is usually more aggressive as patients also have developmental and neurological manifestations, whereas in the later onset form of the disease patients often develop psychiatric features and slowly progressive neurological problems [5],[6].

Prior to the identification of disease genes the diagnosis of NBIA was based on clinical features and ultimately the neuropathological examination, which demonstrated disseminated axonal swellings with various amounts of iron accumulation in structures such as the pallidum and in some cases also in the pars reticulata of the substantia nigra [4],[7]–[9]. As a result of the common finding of axonal swellings in both HSS and iNAD, these conditions were often considered phenotypic variants of the same underlying disorder [4].

The recognition that mutations of the pantothenate kinase 2 (*PANK2*) gene are the commonest cause of NBIA [10], also known as pantothenate kinase-associated neurodegeneration (PKAN), NBIA type 1 or HSS, has significantly advanced our understanding of this disorder [11]. *PANK2* has an essential role in regulating co-enzyme A biosynthesis, catalysing the cytosolic phosphorylation of pantothenate (vitamin B5), N-pantothenoylcysteine and pantetheine and is involved in ceramide metabolism [12],[13]. PKAN is inherited in an autosomal recessive manner and is estimated to be responsible for approximately 50% of all NBIA cases [11]. Patients with PKAN usually have the eye-of-the-tiger sign in the globus pallidus on T2-weighted magnetic resonance imaging (MRI) scans, which consists of a hyperintense central region, corresponding to tissue necrosis or oedema surrounded by a hypointense zone with high iron levels [14]–[16]. Although initially it was thought that there is a close correlation between the eye-of-the-tiger sign and PKAN, it has been recognized that this MRI sign is not pathognomonic for PKAN as it may also be seen in other forms of NBIA [17]. According to the current classification, NBIA type 2 includes iNAD and also juvenile and adult cases with dystonia-parkinsonism syndrome and it has been shown to be associated with mutations in the phospholipase A2, group VI (*PLA2G6*) gene. NBIA is also uncommonly associated with acoeruloplasminaemia due to mutations in the coeruloplasmin (*CP*) gene [18] and neuroferritinopathy associated with mutations in the *FTL* (ferritin light polypeptide) gene [19].

Since the mid-1960s there have been a number of reports describing Lewy bodies in neurones of the pars compacta of the substantia nigra and sometimes in neurones of the locus coeruleus in HSS/NBIA [7],[20],[21]. In addition, it has also been described that some HSS cases may be associated with neurofibrillary tangle pathology [22],[23]. As a result HSS was consistently considered a form of synucleinopathy with potential implications for *PANK2*-related cellular pathways on the pathogenesis of other Lewy body disorders, including Parkinson's disease [24],[25]. Recently, Gregory *et al*. demonstrated that Lewy body pathology is a feature of NBIA type 2 due to mutations of the *PLA2G6* gene [26]. Subsequently, in a detailed neuropathological study of five additional cases with *PLA2G6* mutations, we also confirmed that α-synuclein deposition is a consistent phenomenon in cases with *PLA2G6* mutations and also that the Lewy body pathology may be severe and extensive with a pattern similar to that seen in end-stage Lewy body disorders including dementia with Lewy bodies and Parkinson's disease [27].

The aims of this current study were twofold. First we wished to report the neuropathological features of three previously unreported and genetically proven PKAN cases. Second, in view of the literature data indicating a link between *PANK2* and Lewy body disorders, we wished to establish the incidence of α-synuclein and tau pathologies in this group of cases as, apart from a recent report [28], no other pathological description of genetically proven cases is available in the literature.

## Material and methods

### Material

This project was approved by the Joint Local Research Ethics Committee of the National Hospital for Neurology and Neurosurgery and the UCL Institute of Neurology. Tissue was received from the Brain and Tissue Banks for Developmental Disorders, Baltimore (BTBDD) where brains are obtained and stored with appropriate consent. Clinical details, kindly provided by the BTBDD of three *PANK2* mutation cases. To our knowledge, none of the cases have been previously reported.

### Genetics

Techniques and primer sequences used in this study have been previously reported by our laboratory [27]. In brief, all exons of the *PANK2* gene were sequenced using standard Sanger polymerase chain reaction sequencing on an ABI 3730XL machine (Applied Biosystems, Inc., Foster City, CA, USA) with sequence alignment and repeat analysis in all mutations. The *PANK2* gene was also screened in 100 controls, which were all negative for these variants. *PANK2* primer sequences are in Supplementary Table S1. Additional movement disorder and NBIA genes were also sequenced to rule out possible mutations, these included the *Dyt1*, *PLA2G6* and ferritin light chain genes.

### Neuropathology

Brain regions from three cases with the clinical diagnosis of HSS were available from BTBDD and examined in the laboratory of the Queen Square Brain Bank for Neurological Disorders, using routine histological and immunohistochemical methods. Regions available included frontal and temporal necortex, hippocampus with parahippocampus, amygdala, basal ganglia, midbrain with substantia nigra, pons and medulla. No cerebellar blocks were available for this study. In brief, 7 µm thick sections of paraffin embedded tissue blocks were deparaffinized and rehydrated through graded alcohols. Sections were placed in 0.3% H_2_O_2_ in methanol to block endogenous peroxidase activity. Antigen retrieval of masked antigen epitopes was achieved either by pressure cooking in citrate buffer (pH 6.0) for 10 min, incubation in 99% formic acid for 10 min or incubation in Proteinase K solution for 10 min (see Supplementary Table S2) this was followed by 10% non-fat milk solution to prevent non-specific antibody binding. The appropriate primary antibody was applied (see Supplementary Table S2), followed by an anti-mouse or anti-rabbit secondary antibody (Dako, Ely, UK) as appropriate. Subsequently avidin-biotin complex Elite kit (Vector, Peterborough, UK) was applied and colour was developed by diaminobenzidine/H_2_O_2_. Sections were finally counterstained with Mayer's haematoxylin. In addition, Perls' Prussian blue stain for iron and Gallyas silver staining were performed.

## Results

### Genetics

Case 1 and case 2 were compound heterozygous for the His283Gln and Gly521Arg mutations and the Del423/424Cys and Gly521Arg mutations, respectively. Case 3 was homozygous for the Gly521Arg mutation (Table 1). All three mutations have been described previously and are predicted to be pathogenic according to the sequence change, conservation among lower species and their absence in controls (Table 1).

**Table 1:** *PANK2* gene mutations and clinical features

*Demographics and genetics*							

*Case*	*Sex*	*Ethnicity*	*Age of onset*	*Age at death*	*Nucleotide change*	*Amino acid change*	PANK2 *gene location*
1	Female	American Caucasian	2 years	10 years	Heterozygous 847C>G	His283Gln	Exon 2
					Heterozygous 1561G>A	Gly521Arg	Exon 6
2	Female	American Caucasian	6 months	10 years	Heterozygous Del1267-9	Del423/424Cys	Exon 4
					Heterozygous 1561G>A	Gly521Arg	Exon 6
3	Male	American Caucasian	Childhood	20 years	Homozygous 1561G>A	Gly521Arg	Exon 6

*Clinical details*					

*Case*	*First clinical symptom*	*Ataxia*	*Dystonia*	*Pigmentary retinopathy*	*Main clinical symptoms*
1	Balance problems and falling	Severe later in disease	Yes	Yes	Spasticity, dysarthria progressive rigidity with dyskinesia, dystonia and autistic
2	Developmental delay	Moderate	Yes	Yes	Developmental milestones delayed, weakness, dystonia with hypotonia, impaired co-ordination
3	Details not available	Moderate	Yes	Yes	Rigidity, painful spasms, nystagmus, severe contractures

Del, deletion.

### Clinical data

Table 1 shows a summary of the main clinical data in the three cases. Cases 1 and 2 were diagnosed with iNAD with disease onset at 2 years and 6 months of age, respectively. Case 3 began to show the first symptoms at the age of 10 and was considered to suffer from juvenile NAD.

Case 1 was a Caucasian girl, whose initial development in early infancy was normal, but she developed balance problems and falls at around 2 years of age. Subsequently she showed a delay in her physical development, suffered from increased tone in her limbs, developed dystonia and was found to have pigmentary retinopathy. As a toddler the differential diagnosis also included autism as she had speech delay and by the age of 10 year she had lost all speech. In later life around 6 years of age she developed severe spasticity of her upper and lower limbs with the right side being more affected than the left, progressive rigidity with dyskinesia and dystonia and later she also suffered from seizures. She was unresponsive to Sinemet and Artane. Other symptoms included difficulties with swallowing and chewing (due to bulbar spasticity) and bowel movements. Routine examination and detailed blood and urine screening were negative apart from positive antigliadin antibodies. An MRI scan at 8 years of age was typical for PKAN with the eye of the tiger sign seen on T2 weighted images. Later on she required a gastrostomy tube for feeding her fluids and food. She died at the age of 10 years from respiratory complications of the disorder.

Case 2 was a Caucasian girl, who at 6 months of age was found to be weak and unable to roll over or crawl. At 15 months she was diagnosed with ‘mild cerebral palsy’, spasticity and dystonia. At the age of 2 years she began to have visual problems and was found to have pigmentary retinopathy. She was documented to be underweight and showing rigidity, dystonia with hypertonia and impaired co-ordination at the age of 4 years. She had two unaffected siblings; however, there was a paternal second cousin with ‘amyotrophic lateral sclerosis’-like neurological problems and the maternal grandmother had parkinsonism. She was extensively investigated, but blood and CSF did not show any abnormalities. An MRI scan showed increased signal in the globus pallidus with a hypointense periphery (‘the eye of the tiger sign’) on T2 weighted images, but sparing of the substantia nigra. Electroencephalogram showed low background with low voltage but no epileptiform discharges. Electroretinogram showed no rod and cone discharges and was essentially flat. At the age of 9 she was documented to have uncontrollable writhing movements, dysarthria, seizures, and difficulty with swallowing, rigidity, spasticity and spasms in all extremities and tremor. A number of treatments were tried including Prednisolone, L-Dopa, Artane, Carbatrol, Selegiline and Baclofen without any benefit. Subsequently her condition further deteriorated and she developed dysarthria and feeding difficulties requiring a gastrostomy tube. Towards the end of her life she suffered episodes of respiratory insufficiency with aspiration pneumonia.

Case 3. This Caucasian boy presented in late childhood with dystonia and rigidity, although no information is available about the exact age when his first symptoms began. At the age of 10 years he had an MRI scan, which showed a hypointense globus pallidus and a hyperintense area anterior and medial to the globus pallidus indicative of ‘the eye of the tiger’ sign seen on T2 weighted images. He responded poorly to medication and by the age of 11 years his movement disorder was severe enough to warrant a left pallidotomy, followed by a right pallidotomy a year later, to relieve the main features of severe dystonia and painful muscle spasms and rigidity. Despite surgery and medical therapy he continued to deteriorate. Two months before death he was noted to have very severe contractures, reduced facial expression although he would smile on some occasions, pigmentary retinopathy in both fundi and nystagmus on horizontal gaze. His reflexes were difficult to obtain due to the severe contractures and his plantars were unresponsive. He was admitted to and died in hospital with ‘double pneumonia’ a few weeks short of 21 years of age.

### Neuropathology

#### Macroscopic findings

In all three cases the weight of the unfixed brains was in the normal range (case 1: 1380 g; case 2: 1225 g; case 3: 1330 g). The leptomeninges and the arteries of the circle of Willis, the major arterial branches and the superficial veins were unremarkable. There was no cerebral or cerebellar cortical atrophy. At brain slicing the cortical ribbon was well preserved and the ventricular system was unremarkable. The globus pallidus showed rusty discolouration and reduction in size in all three cases. Sections of the brainstem showed pale substantia nigra and locus coeruleus consistent with age.

#### Histological findings

Table 2 summarizes the main pathological features of the three PKAN cases. As there were only quantitative differences in the microscopic appearances between the cases, the microscopic findings are described together.

**Table 2:** Semiquantitative summary of tau pathology, axonal swellings and gliosis in three cases with pantothenate kinase-associated neurodegeneration

	*Case 1 (4561)*			*Case 2 (4733)*			*Case 3 (5097)*		
			
*Brain region*	*Tau*	*AS*	*Gliosis*	*Tau*	*AS*	*Gliosis*	*Tau*	*AS*	*Gliosis*
Frontal neocortex	−	+	++	−	−	+	+++	−	++
Temporal neocortex	−	+	+	−	+	+	+	−	++
Globus pallidus	−	+++	+++	−	+	++	−	+	+++
Putamen	−	++	++	−	+	++	−	−	+++
Putamen pencil fibres	−	++	−	−	++	−	−	−	−
Caudate	−	+	++	−	+	++	−	−	+++
Caudate pencil fibres	−	++	−	−	+	−	−	−	−
Meynert nucleus	−	−	−	−	−	−	+	+	++
Hippocampus	±*	+	++	−	+	++	−	+	+
Subiculum	−	+	+	−	−	+	−	−	−
Entorhinal/transentorhinal cortex	−	−	+	−	+	+	+++	−	+
Fusiform gyrus	−	−	+	−	+	+	+	−	+
Amygdala	N/A	N/A	N/A	−	−	−	+*	+	+
Substantia nigra	−	+	−	−	+	−	+	+	+
Pons	−	−	−	−	−	+	−	++	+
Cuneate and gracile nuclei	−	+	−	−	−	−	−	+	+

The severity of tau pathology, axonal swellings and gliosis was assessed semi-quantitatively.

* Tau positive neuropil threads are present, but no neurofibrillary tangles are seen.

−, absent, +, occasional/mild, ++, moderate, +++, severe.

AS, axonal swellings; N/A, not available.

**Figure 1:**
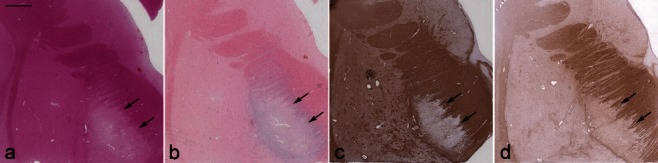
Involvement of the globus pallidus (arrows) in neurodegeneration with brain iron accumulation type 1 due to mutation of the *PANK2* gene. Rarefaction of the neuropil (**a**) is accompanied by iron deposition (**b**), myelin (**c**) and axonal loss (**d**). (**a**) Haematoxylin and eosin, (**b**) Perls' iron, (**c**) myelin basic protein (CD94) immunohistochemistry and (**d**) phosphorylated neurofilament heavy chain (RT97) immunohistochemistry. Bar on a represents 4000 µm on **a**–**d**.

**Figure 2:**
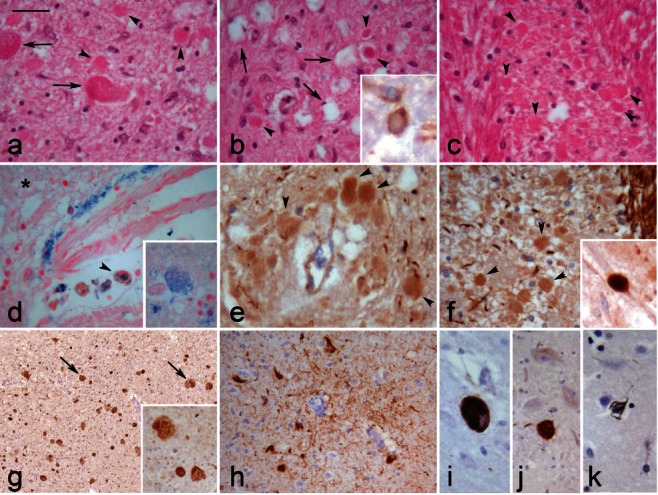
Main microscopic findings in neurodegeneration with brain iron accumulation (NBIA) type 1 are illustrated by the findings in case 3. Neuroaxonal dystrophy is centred on the globus pallidus and basal ganglia. (**a**–**c**) Axonal swellings ranged from relatively small (arrowheads) to large structures (arrows), which are reminiscent of the ovoid bodies in superficial siderosis. (**b**) In the globus pallidus small axonal swellings (arrowheads) are seen to be surrounded by a thin layer of myelin (insert). Large vacuoles (arrows) seen in the close vicinity of axonal swellings are likely to represent resorption of swellings. Significant deposition of iron in the globus pallidus is a constant feature. Diffuse, dust-like deposition of iron pigment (asterisk) while the pigment is coarse elsewhere including in perivascular macrophages (arrowhead). The insert on **d** demonstrates iron deposition in a typical ovoid body. (**e,f**) Axonal swellings (arrowheads) were immunoreactive for phosphorylated neurofilament heavy chain and amyloid precursor protein (insert on **f**). (**g** and insert on **g**) The large axonal swellings (ovoid bodies) (arrows) are strongly immunoreactive for ubiquitin and can be numerous in the globus pallidus in NBIA type 1. Tau immunohistochemistry demonstrates a cluster of neurofibrillary tangles and a dense meshwork of neuropil threads in the frontal cortex (**h**), a neurofibrillary tangle in the Meynert nucleus (**i**) and substantia nigra (**j**). The neurofibrillary tangles were Gallyas silver-positive (**k**). (**a**–**c**) Haematoxylin and eosin, (insert on **b**) myelin basic protein (CD94) immunohistochemistry, (**d**) Perls' preparation for iron, (**e**) SMI31 immunohistochemistry, (**f**) RT97 immunohistochemistry, (insert on **f**) amyloid precursor protein immunohistochemistry, (**g**) ubiquitin immunohistochemistry, (**h**–**j**) tau (AT8) immunohistochemistry, (**k**) Gallyas silver impregnation. Bar on **a** represents 30 µm on **a**–**d**, **f**, **h**–**k**, insert on **g** and 20 µm on **e**, inserts on **b**, **d**, **f**.

The neocortical hexalaminar architecture was preserved throughout, although there was astrogliosis in neocortex, entorhinal cortex and also subcortical white matter in all three cases. These changes were of moderate degree in both the frontal and temporal regions in case 3 and were mild in cases 1 and 2. There was marked astrogliosis in the hippocampal end-folium in case 1, which was mild in cases 2 and 3. Involvement of the globus pallidus was the major finding in all cases. In case 1 there was marked myelin pallor, axonal loss (Figure 1**a**,**c**,**d**) and prominent vacuolation of the neuropil together with a significant increase in number of reactive astrocytes and foamy macrophages in some of the vacuoles (Figure 2**b**). In case 2 the morphological abnormalities of the globus pallidus were similar, but considerably milder while in case 3 these were very severe with cavitation involving both the internal and external segments of this nucleus surrounded by a significant degree of astrogliosis. Loss of globus pallidus neurones of various degrees was seen in all three cases (case 3 > case 1 > case 2). The extent of the iron deposition, which was most severe in case 1, was best appreciated on Perls' stain utilizing the Prussian blue reaction (Figure 1**b**). This also showed that iron pigment was associated with astrocytes and microglia and was also seen to accumulate in macrophages, mostly around small blood vessels (Figure 2**d**). The presence of diffuse, dust-like haemosiderin deposition, finely impregnating the neuropil of the globus pallidus was also a feature (Figure 2**d**). Varying numbers of large eosinophilic structures with a granular appearance, measuring between 20 µm and 70 µm and most numerous in case 1 (Figure 2**a**), were seen in all cases (case 1 > case 3 > case 2). Overall the morphological appearances of these structures were similar to those described as ovoid bodies in superficial siderosis. These structures were strongly ubiquitin-positive often with a granular staining pattern, but to a lesser extent for p62 (Figure 2**g**). Occasional ovoid bodies were neurofilament-positive. Perls' preparation demonstrated that a proportion of the ovoid bodies showed either a weak diffuse or a more granular staining pattern (Figure 2**d**, insert). The presence of axonal spheroids, the size of which varied between 10 µm and 30 µm, was a prominent feature of all three cases, although there was some variation in their numbers between the cases (case 1 > case 2 > case 3). They were most numerous in the globus pallidus and were also readily found in the medial putamen, bordering the external pallidum, in the pencil fibres of the striatum and the grey matter bridges interconnecting the putamen and caudate nucleus (Figure 2**b**,**c**). In our cases the axonal spheroids were best demonstrated with phosphorylation dependent anti-NF-H antibodies (RT97 and SMI31) (Figure 2**e**,**f**), although the intensity of their staining was somewhat variable. They were also seen on the APP immunohistochemical preparations (Figure 2**f**, insert). In contrast to ovoid bodies, axonal spheroids were weakly ubiquitin-positive. Myelin basic protein immunohistochemistry demonstrated loss of myelin sheaths in the pallidum and that small axonal swelling were surrounded by a thin layer of myelin (Figures 1**c** and 2**b**, insert).

There was no cell loss in the pars compacta of the substantia nigra or other brainstem nuclei in the midbrain, pons or medulla in the three cases studied. In particular, α-synuclein immunohistochemistry, carried out using three different antibodies on tissue sections of cerebral cortex, basal ganglia with the Meynert nucleus, brainstem, including substantia nigra and locus coeruleus was entirely negative. However, in case 3 tau immunohistochemistry using the phosphorylation-dependent AT8 anti-tau antibody recognizing Ser202/Thre205, demonstrated neurofibrillary tangles and neuropil threads in the frontal cortex and pre-α neuronal clusters of the entorhinal cortex. An occasional neurofibrillary tangle was also seen in the temporal neocortex, and the Meynert nucleus (Figure 2**h**,**i**). Tau isoform-specific immunohistochemistry showed that tau in the neurofibrillary tangles and neuropil threads were composed of both 3-repeat and 4-repeat tau isoforms. Both the neurofibrillary tangles and neuropil threads were also labelled with the AT100 anti-tau antibody and were ubiquitin, p62 and Gallyas silver-positive (Figure 2**k**). In case 1 an occasional tau-positive neuropil thread was noted in the hippocampus. None of the cases had any Aβ peptide deposition in parenchyma or blood vessels.

## Discussion

In this comparative study we have provided clinical, genetic and pathological data for three cases with PKAN/NBIA type 1. Our cases 1 and 2 had a classical clinical presentation whereas case 3 presented later in life with a dystonic rigid picture consistent with juvenile NAD [11],[13]. As there is a significant overlap between NBIA type 1 and type 2 cases in clinical presentation, both the *PLA2G6* and *PANK2* genes were sequenced, which showed that all three cases are associated with mutations of the *PANK2* gene. These were compound heterozygous mutations in cases 1 and 2 and homozygous in case 3. All three mutations are predicted to be pathogenic and were not present in our cohort of appropriate controls.

In keeping with the classical description of PKAN/NBIA type 1 neuropathological examination of all three cases demonstrated axonal spheroids and neuronal cell loss with parenchymal damage of the neural parenchyma of the globus pallidus. Deposition of Perls' positive iron pigment and the presence of various numbers of ovoid bodies were also constant features. Axonal swellings, which in this study were best demonstrated by high molecular weight phosphorylated neurofilament immunohistochemistry and showed variable reactivity to ubiquitin and APP, were most numerous in the globus pallidus, followed by the medial putamen, including pencil fibres adjacent to the globus pallidus and grey matter bridges of the striatum interconnecting the putamen with the caudate nucleus. In another, recently reported series of genetically confirmed PKAN cases the neuropathological changes were also largely centred on the globus pallidus and surrounding structures [28]. This observation is in contrast with the rather widespread distribution of neuroaxonal swellings that is present in NBIA type 2 with *PLA2G6* mutations, in which brain iron accumulation is rather variable and axonal swellings are frequent in the globus pallidus, brainstem nuclei as well as spinal cord and cerebellar degeneration may also be a prominent feature [26],[27]. Axonal swellings are a central feature of both NBIA type 1 and type 2, although the mechanisms leading to their formation remain to be understood. Axonal swellings are thought to be due to defects in axonal transport or membrane integrity, which in NBIA type 2 is likely to be related to the critical role calcium-independent PLA2 enzymes play in cell membrane homeostasis [29]. As no pathological involvement of the basal ganglia can be seen in the *PANK2* knock-out mice model so far reported [30], our understanding of how neuroaxonal swellings are formed in NBIA is largely reliant on data from *PLA2G6* knock-out mouse models [31],[32]. These studies confirm that axonal swellings arise from degeneration of swollen axons or synaptic terminals while the large vacuoles, which were seen in our PKAN cases and have also been described in the *PLA2G6* knock-out animal models, are thought to occur as a result of resorption of spheroids via a cellular process involving ubiquitination [32]. The relevance of the axonal spheroids for clinical disease is underpinned by the data indicating that in the *PLA2G6* mouse model the onset of motor impairment correlates with a significant increase in number of the ubiquitin-positive spheroids in nearly all brain regions [31].

An electron microscopic study of neuroaxonal dystrophies has demonstrated two types of axonal changes in HSS according to the size and ultrastructural composition of the axonal swellings [33]. According to this study the size of the axonal swellings varied between 20 µm and 70 µm. The ‘small’ axonal swellings are surrounded by a myelin sheath, which is also confirmed by our light microscopy study, and are composed of dense and vesicular bodies, degenerating mitochondria and amorphous material. The ‘large’ axonal swellings, measuring up to 70 µm, are not surrounded by a myelin sheath and contain predominantly dense bodies and abnormal mitochondria [33]. Similar to a recent study of PKAN cases by Kruer *et al*. [28] we were also able to confirm the presence of often numerous eosinophilic, ubiquitin-positive structures in the globus pallidus affected by iron deposition, which correspond to the ‘large’ dystrophic axons described and illustrated by Malandrini *et al*. [33]. In our cases the size of the majority of these structures varied between 20 µm and 40 µm with a few measuring up to 70 µm. Their morphological features are identical to the ‘ovoid bodies’ described in association with severe haemosiderin deposition such as that seen in superficial siderosis or in the vicinity of cavernous haemangiomas accompanied by chronic or repetitive haemorrhage [34]. The similarity in this aspect of the pathology between diverse conditions such as NBIA and superficial siderosis indicates that ‘ovoid bodies’ can occur irrespective of whether the iron overload is blood derived or it is due to abnormal iron homeostasis such as that associated with PKAN. The notion that such structures may be seen in the absence of blood derived iron deposition is further supported by the finding of structures with morphological appearances similar to ‘ovoid bodies’ in various forms of Parkinsonian disorders such as Parkinson's disease and progressive supranuclear palsy with degeneration of the substantia nigra and significant iron deposition (data not shown). As far as the origin of these structures is concerned Kruer *et al*. [28] suggested that such large granular and ubiquitin-positive structures (‘ovoid bodies’) represent degenerating neurones. As an alternative hypothesis an axonal origin of these structures was previously proposed, which would be supported by their description in the crus cerebri or the eighth cranial nerve affected by severe haemosiderin deposition in superficial siderosis [34],[35]. Further detailed studies are required to establish the origin of the ‘ovoid bodies’ and their relationship to the cellular mechanisms involved in iron regulation in different forms of NBIA.

In view of the importance of the suspected link between PANK2 and α-synuclein aggregation and Lewy body formation [24] and the potential association of PANK2 with tau aggregation, one of the major aims of our study was to investigate whether Lewy body or neurofibrillary tangle pathology is present in any of our genetically proven PKAN cases. Since the discovery that distinct genetic abnormalities define the two major forms of NBIA presenting with NAD, cases with *PLA2G6* mutations have been shown to be closely associated with often severe, α-synuclein and tau pathologies [26],[27]. However, it has remained uncertain whether NBIA type 1 cases with mutations in the *PANK2* gene would also show a similar association. The information provided by the recent study of Kruer *et al*. [28] and by our current study now clearly indicates that Lewy body pathology is not a feature of PKAN/NBIA type 1. It is of interest that we were able to confirm focal tau pathology with neurofibrillary tangles and neuropil threads in cerebral cortex, anterior temporal mesocortex and the Meynert nucleus in our case 3, who had a later disease onset and died at the age of 20 years. In addition, occasional neuropil threads were seen in case 1. Not only were the neurofibrillary tangles and neuropil threads stained with the phosphorylation-dependent AT8 antibody in our case 3, but they were also labelled with the 3-repeat and 4-repeat tau-specific antibodies indicating that, as in paired helical filament tau, both major classes of tau isoforms contribute to the tau inclusions in our PKAN case. Furthermore, the neurofibrillary tangles and neuropil threads were also labelled with the AT100 anti-tau antibody and stained by the Gallyas silver method, indicating filamentous tau pathology [36]. The fact that case 3 was the oldest in our series may be important and indicate that age is an important factor in the initiation of NBIA-associated tau pathology. It is of note that this is in contrast to our previously reported observation indicating that in NBIA cases associated with mutations in the *PLA2G6* gene, the tau pathology, which was severe in three of the five pathologically studied cases, tended to be less severe in the later onset cases while all cases had severe Lewy body pathology [27].

Our study further defines the clinical spectrum and neuropathology of *PANK2*-associated NBIA type 1 and provides a comparison to the NBIA type 2 cases associated with *PLA2G6* mutations. The clinical phenotype of the cases with *PANK2* and *PLA2G6* is remarkably similar in the initial stages of the two diseases when it would be very difficult to differentiate the two as both have developmental delay, spasticity and dystonia. Later in the disease course, there are clinical differences with *PLA2G6* cases having ataxia, greater Parkinsonian features and a better response to L-Dopa. Imaging is also a way to differentiate the two with *PANK2* having the eye of the tiger sign on MRI while *PLA2G6* cases show the hypodense basal ganglia signal. Importantly our current study has demonstrated that Lewy body pathology is not a feature of NBIA type 1, which is in contrast to previous confirmation of severe α-synuclein pathology in NBIA type 2. The presence of abnormal tau pathology could suggest an important aetiological role of *PANK2*, which has been implicated in mitochondrial function, iron and tau [37].

## Supporting information

**Table S1.** Oligonucleotide primers used toamplify the exonic regions and flanking introns of thephospholipase A2, group VI (*PLA2G6*), the pantothenate kinase2 (*PANK2*) and the ferritin light chain (*FTL*)genes.

**Table S2.** Antibodies used for immunohistochemistry investigation.
