# Supplementary material for: Pantothenate kinase-associated neurodegeneration is not a synucleinopathy
Source: Neuropathol Appl Neurobiol. 2012 Mar 14;39(2):121–31. doi: 10.1111/j.1365-2990.2012.01269.x (PMC3712463; doi:10.1111/j.1365-2990.2012.01269.x)
Supplement: Supplementary file 1 [file nan0039-0121-SD1.doc]

Supplementary Table 1. Oligonucleotide primers used to amplify the exonic regions and flanking introns of the Phospholipase A2, group VI (PLA2G6), the Pantothenate Kinase 2 (PANK2) and the Ferritin Light Chain (FTL) genes

| **PLA2G6** | **Forward sequence (5’ to 3’)** | **Reverse sequence (5’ to 3’)** |
| --- | --- | --- |
| Exon 2 | GTGTCTGTGCAGGAAACCG | GCCAATAAGACCTCCAATCC |
| Exon 3 | TGATTCCAGCAGGGATGTG | AACTATGGAGGGGAACCGAG |
| Exon 4 | AAAGTCCGAGTTTCCGAGTG | AGGCCTGAGAGTGACACCTG |
| Exon 5 | GTGATCCACCCACCTTGG | TGGTGGATACTGCTTGCCTC |
| Exon 6 | CTTCATCCCACGCCACG | GAACCTGCTTCCTGAGGG |
| Exon 7 | TCAGAGCAGAAGTGGCAGTG | GGGAGGAGGGCTCCAGTC |
| Exon 8 | CTGGGTGAGTTGACAGGTTG | ACTTCCCTCCTCCTCGGTC |
| Exon 9 | AGTGTGGAAAGGAGGGGC | GATCCTGTTGCTTTGGTGG |
| Exon 10 | CTAGGGACCTCTGGGGTAGC | GTGAGGGGCAGGAAAGC |
| Exon 11 | ACAAGGGCTATGAGGGTGG | GCAAAGCCCTGAAGACAAAC |
| Exon 12 | GCTCTGCAGGCTGTTCTACG | CTCAGCAGGACAGGGAGC |
| Exon 13 | GTGTGAATTGTGGGGAAAGG | GATGGCAAGTGCACGACTC |
| Exon 14 | CTGAGATCTGGAGTGCATGG | GTCCCTAGCATGGTTTGCTG |
| Exon 15 | CCCCAGAGCCCAGTCTTG | AGGATGAGGGGAAGCCATC |
| Exon 16 | CTGACTCGAAAGAGCCTGG | GGGAACAGAGCAGACCCTTG |
| Exon 17 | ACCCTGGTCCTAGCTGGC | GGCAGGGGTACGGTTGTG |
| **PANK2** | **Forward sequence (5’ to 3’)** | **Reverse sequence (5’ to 3’)** |
| Exon 1A | GCTCTATTCCAGAGACCGAGTG | ATTTCCAACTTGAAATCTAACCAG |
| Exon 1B | ACCAGCCTGGACAACATAGTG | GACTGGAACAGAATTCAACTGAG |
| Exon 2 | TTTCAGCACTTAGTTCACTTTAGG | CCAGAACTTCACCAATATAGCAC |
| Exon 3 | TTATTAAGAGGACTGTGTGGAGTG | CTCATATTCCATGATCTTCCAGAC |
| Exon 4 | TTTACTTCATGTGATGCCAGG | TCTTAAACAAACCACATTGTCTTC |
| Exon 5 | GCACTGTACTTCTTCCATGAGG | CAGTCAGATGTCATACTCACCAAG |
| Exon 6 | TTGTTGTAGATGATGCATACTTGG | AGAGTTTTAGGGACACAGGCAC |
| Exon 7 | ACTGTTTAATGCAGGACGAATG | GATGACTACTCCAGCACAGACAAC |
| **NFL** | **Forward sequence (5’ to 3’)** | **Reverse sequence (5’ to 3’)** |
| Exon 4 | GCCTCATTTCACACCTGTC | CTCCTCTTTCACTGGCATC |
