# Supplementary material for: Pantothenate kinase-associated neurodegeneration is not a synucleinopathy
Source: Neuropathol Appl Neurobiol. 2012 Mar 14;39(2):121–31. doi: 10.1111/j.1365-2990.2012.01269.x (PMC3712463; doi:10.1111/j.1365-2990.2012.01269.x)
Supplement: Supplementary file 2 [file nan0039-0121-SD2.doc]

Supplementary Table 2. Antibodies used for immunohistochemistry investigation

| **Antibody** | **Source** | **Species** | **Antigen retrieval** | **Dilution** | **Incubation time** |
| --- | --- | --- | --- | --- | --- |
| Ubiquitin | Dako, Ely, UK | Rabbit | CB, PC | 1:200 | 1 hour |
| P62 | BD Biosciences, Oxford UK | Mouse | CB, PC | 1:100 | 1 hour |
| AT8 | Autogen Bioclear, Witshire, UK | Mouse | CB, PC | 1:600 | 1 hour |
| AT100 | Innogenetics, Gent, Belgium | Mouse | CB, PC | 1:200 | 1 hour |
| 3-repeat tau | Courtesy of Dr Rohan dr Silva | Mouse | CB, PC | 1:10,000 | 1 hour |
| 4-repeat tau | Courtesy of Dr Rohan dr Silva | Mouse | CB, PC | 1:200 | 1 hour |
| α-Synuclein | Novocastra, Newcastle Upon Tyne, UK | Mouse | CB, PC, FA | 1:50 | 1 hour |
| α-Synuclein | BD Transduction Biolabs, Oxford, UK | Mouse | FA | 1:1000 | Overnight |
| α-Synuclein (S129) | Abcam, Cambridge, UK | Rabbit | CB, PC, FA | 1:1000 | Overnight |
| Amyloid-β peptide | Dako, Ely, UK | Mouse | CB, PC, FA | 1:200 | 1 hour |
| APP | Chemicon, Watford UK | Mouse | CB, PC | 1:100 | 1 hour |
| TDP-43 | Protein Tech, Manchester, UK | Rabbit | CB, PC | 1:2000 | 1 hour |
| GFAP | Dako, Ely, UK | Rabbit | PK | 1:1000 | 1 hour |
| CD68 | Dako, Ely, UK | Mouse | CB, PC | 1:150 | 1 hour |
| IBA1 | Wako Chemicals, Hampshire, UK | Rabbit | CB, PC | 1:1000 | 1 hour |
| Neurofilament cocktail | Cappell, Loughborough, UK | Mouse | None | 1:20 | 1 hour |
| RT97 | Novocastra, Newcastle Upon Tyne, UK | Mouse | CB, PC | 1:50 | 1 hour |
| SMI31 | Sternberger Monoclonals, Leeds, UK | Mouse | CB, PC | 1:2000 | 1 hour |
| SMI32 | Sternberger Monoclonals, Leeds, UK | Mouse | CB, PC | 1:2000 | 1 hour |

*CB* citrate buffer, *PC* heat pretreatment in pressure cooker, *FA* formic acid, *PK proteinase* K, *APP* amyloid precursor protein
